# 3.75 THz Repetitive Radiation Regulates Collagen Metabolism in Human Fetal Scleral Fibroblasts

**DOI:** 10.3390/cells15141285

**Published:** 2026-07-17

**Authors:** Wenxia Wang, Liu Sun, Lei Wang, Jinwu Zhao, Pandeng Hou, Mingxia He

**Affiliations:** 1 The Center for Terahertz Waves, School of Precision Instrument and Opto-Electronics Engineering, Tianjin University, Tianjin 300072, China; 2 State Key Laboratory of Precision Measuring Technology and Instruments, Tianjin University, Tianjin 300072, China

**Keywords:** human fetal scleral fibroblasts, terahertz radiation, collagen metabolism, myopia, proteomics analysis

## Abstract

The sclera constitutes a critical structural element of the eyeball, as its biomechanical properties and structural integrity are primarily governed by collagen metabolism. Using a controlled in vitro experimental system, the present study explored the quantitative relationship between exposure duration to terahertz radiation of distinct frequencies (3.10 THz and 3.75 THz) and the transcriptional expression of collagen metabolism-associated genes in human fetal scleral fibroblasts (HFSFs). During the 120 min terahertz irradiation exposure, neither of the two frequencies produced significant regulatory effects on collagen metabolism-associated genes in normal and hypoxia-induced HFSFs. However, 3.75 THz radiation had markedly raised the expression level of the COL1A1 gene in HFSFs 12 h after the terahertz radiation stopped. Proteomic analysis was conducted on scleral fibroblasts exposed to repeated radiation at 3.75 THz. Proteomic analysis identified a total of 213 differentially expressed proteins (DEPs), comprising 172 downregulated proteins and 41 upregulated proteins. These DEPs were predominantly enriched in ribosome-associated proteins and mitochondrial function-related proteins, suggesting a potential regulatory role in intracellular protein synthesis and ATP production. Furthermore, the study demonstrated that expression levels of fourteen proteins associated with the collagen-rich extracellular matrix exhibited a marked enrichment trend, indicating that repeated exposure to 3.75 THz radiation induces remodeling changes in the scleral extracellular matrix (ECM). This study demonstrates the impacts of terahertz radiation on collagen metabolism of HFSFs and provides preliminary molecular evidence, yet direct functional effects remain unconfirmed. These findings provide theoretical and experimental bases for studying the regulation of scleral collagen metabolism by terahertz radiation.

## 1. Introduction

Myopia has become a significant worldwide public health concern [1]. Current myopia control techniques are severely limited due to the intricacy of the etiology of myopia. The majority of myopic patients have axial myopia, which is characterized by abnormalities such as severe axial eye elongation and scleral thinning [2]. When myopia develops, a number of neurotransmitters and growth factors are released by retinal cells. In response to these signaling molecules, scleral fibroblasts alter the remodeling of extracellular matrix proteins [3]. Collagen synthesis and degradation work together to produce the dynamic process of scleral remodeling [4]. Reduced amounts of collagen and glycosaminoglycans (GAGs) and higher levels of matrix metalloproteinases (MMPs) are characteristics of scleral remodeling, as shown in mammalian models of myopia [5]. In the clinical therapy of myopia, it is crucial to find safer and more efficient treatments for these evolving pathologic characteristics in the myopic sclera.

Currently, the main interventions for myopia control include low-dose atropine administration and corrective spectacle wear. Nevertheless, debates still persist regarding their ability to effectively slow down myopia progression and the appropriate timing for treatment cessation, as well as their long-term therapeutic effects [6,7]. With the rapid advancement of interdisciplinary research bridging physics and biology, the modulation of physiological activities in organisms via physical approaches (including ionizing and non-ionizing radiation) has gradually evolved into a key research direction across optics and life sciences. Scleral fibroblasts represent the predominant cell type in the sclera and regulate extracellular matrix remodeling through collagen synthesis and degradation. As the sclera is the ultimate pathological target tissue in myopia progression, scleral fibroblasts have become the most widely used experimental model for exploring scleral functional alterations. Notably, previous studies investigating the biological effects of different types of radiation on scleral fibroblasts have yielded inconsistent and highly variable results. For example, beta radiation can, in a dose-dependent manner, inhibit the proliferation of human Tenon’s fibroblasts, and greater doses of beta radiation result in fewer experimental cells [8,9,10,11]. The mRNA expression of extracellular matrix genes is downregulated by outdoor UVA levels (365 nm, 3 mW/cm^2^), which also decrease collagen synthesis and TGF-β signaling [11]. In HFSF, exposure to extremely low-frequency electromagnetic fields (ELF-EMF) increases the expression level of matrix metalloproteinase-2 (MMP-2) and decreases the expression level of collagen type I (COL1A1) [12,13]. These results indicate that physical interventions based on ionizing radiation generally exert negative regulatory effects on scleral fibroblasts, while photobiomodulation (PBM) produces positive regulatory impacts. Under hypoxic conditions, PBM at 660 nm prevents human scleral fibroblasts (HSFs) from undergoing apoptosis and stimulates collagen synthesis by suppressing the expression of hypoxia-inducible factor-1α (HIF-1α) [14]. Although several recent studies have verified that PBM is capable of slowing myopia progression, research in this field is still relatively scarce. Concerns about its long-term safety after irradiation and challenges related to clinical translation have impeded large-scale research efforts [15,16]. In summary, collagen synthesis in scleral fibroblasts can be modulated by radiation with specific frequencies and power intensities. Nevertheless, the aforementioned physical stimuli are still insufficient to support long-term clinical intervention for myopia. There is an urgent demand for safer and more effective therapeutic strategies to reverse or alleviate progressive pathological remodeling of the myopic sclera in clinical myopia management.

Electromagnetic waves having a frequency between 0.1 and 10 THz (1 THz–10^12^ Hz) are known as terahertz waves. They do not have enough energy to harm DNA or other biological molecules by ionization. Growing interest in the biomedical uses of terahertz radiation is a result of the advancement of terahertz technology and the extensive use of terahertz sources. Terahertz radiation can influence biological systems through non-thermal effects mediated by DNA-directed gene transcription, as shown by numerous studies in the fields of stem cell differentiation [17,18,19], wound response [20], immunomodulation [21,22], and neuromodulation [23,24]. Furthermore, the cells themselves were not negatively impacted by the majority of THz intensities used in these investigations. In human ARPE-19 retinal pigment epithelial cells, our group has shown that a broadband terahertz source can enhance the enrichment of genes associated with the extracellular matrix and cause an increase in COL1A1 gene expression [25]. Additionally, the TGF-β/Smad signaling pathway in HFSFs can be activated by 0.10 THz radiation [26]. It has been shown that transforming growth factor-β (TGF-β) is essential for controlling the extracellular matrix’s turnover. Based on these findings, we hypothesize that terahertz radiation may regulate collagen metabolism in scleral fibroblasts by altering the expression of these collagen-related genes. This study aimed to elucidate the regulatory mechanisms underlying the effects of terahertz radiation physical parameters on cellular functions.

In current research regarding the regulation of biological functions by terahertz radiation, parameter settings including radiation frequency, power, and irradiation duration differ greatly, which further causes discrepancies in terahertz-induced biological effects among different studies. The frequencies selected for terahertz radiation are mainly concentrated in the range of 0.1–3.0 THz, and research reports on the regulatory effects of terahertz radiation at higher frequencies on biological functions are relatively insufficient. Currently, research concerning the frequency-dependent biological effects of terahertz radiation is severely limited, and the patterns underlying its differential regulatory impacts on biological functions as well as their corresponding mechanisms remain poorly understood. The power densities adopted in current studies also cover a wide range from several microwatts (μW) to hundreds of milliwatts (mW). High-intensity terahertz radiation exerts prominent thermal effects, which can induce skin damage and inflammatory responses within only a few minutes of irradiation [27]. In contrast, low-intensity terahertz radiation with a power density lower than 1 mW/cm^2^ can modulate the expression of intracellular biological macromolecules such as genes and proteins to alter cellular biological behaviors, without causing cellular DNA damage, thereby exhibiting favorable biological safety [28,29]. In terms of the selection of terahertz irradiation duration parameters, exposure time varies from several minutes to multiple days across existing studies. In studies on the effects of terahertz radiation on the nervous system, short-term terahertz irradiation has been shown to induce changes in neuronal membrane potential, membrane permeability, and neurotransmitter levels [30,31]. By contrast, one study investigating the relationship between 33 THz irradiation and telomerase activity found that exposure to terahertz radiation significantly suppressed telomerase activity in cancer cells, with this inhibitory effect becoming more pronounced as irradiation time was extended [32]. For long-duration terahertz treatments, 14-day 33 THz irradiation ameliorates cognition and stabilizes neural homeostasis in dementia mice [33], whereas three months of 0.14 THz intervention rescues cognitive deficits and mitigates Alzheimer’s disease neuropathology in AD model animals [34]. These results suggest that terahertz-induced early molecular changes must undergo prolonged intracellular signaling cascades for effect amplification before significant, detectable differences emerge at the cellular and tissue levels. In summary, when selecting terahertz irradiation parameters in future studies, it is recommended to use high-frequency, low-power, and long-duration exposure modes in order to systematically analyze the diverse biological effects of this radiation at the cellular and tissue levels.

Studies investigating terahertz radiation and skin tissues have shown that such radiation can regulate biological processes, including wound healing and remodeling of extracellular matrix components. Given the analogous properties of scleral and cutaneous tissue, the sclera is readily exposed to ambient terahertz radiation. Human fetal scleral fibroblasts (HFSFs) were therefore employed in this study to elucidate the mechanistic impacts of terahertz irradiation on cell viability, collagen turnover, and associated signaling cascades. To explore the frequency-dependent effects of terahertz radiation on cells, two frequencies of 3.1 THz (radiant power: 0.26 mW) and 3.75 THz (radiant power: 0.28 mW) were adopted in this study for irradiation treatment. To evaluate the biosafety of low-frequency terahertz irradiation, three exposure durations (60 min, 120 min, and 180 min) were established, and the changes in cell viability of HFSFs under 3.75 THz irradiation were detected. To investigate the effects of 3.1 THz and 3.75 THz terahertz irradiation on collagen metabolism in HFSF cells, we selected COL1A1, MMP-2, TIMP-2, and TGF-β2 as biomarkers. We further analyzed three aspects: immediate responses during radiation, hysteresis effects following radiation, and radiation responses of hypoxia-induced HFSF cell models. To investigate the immediate responses of gene expression during irradiation, irradiation durations of 30 min, 60 min, and 120 min were set. Hysteresis effects induced by 120 min terahertz irradiation were evaluated 12 h after the irradiation treatment concluded. HFSF cells were incubated in a hypoxic atmosphere (1.5–3% O_2_) for 12 h to generate a myopia pathological cell model. Changes in the expression of four genes were evaluated after exposing the hypoxic model to 120 min of terahertz radiation at two different frequencies. Based on the above experimental results, the optimal terahertz irradiation parameters for regulating collagen metabolism in HFSFs were screened out. After evaluating the growth status of HFSFs cultured in 24-well plates, an irradiation protocol consisting of 120 min daily exposure for five consecutive days was adopted, followed by subsequent proteomic analysis. Therefore, this work may provide references for the selection of terahertz irradiation parameters to modulate collagen metabolism in scleral fibroblasts and could offer valuable experimental evidence for regulating scleral metabolism associated with myopia.

## 2. Materials and Methods

### 2.1. Cell Culture

The human fetal scleral fibroblasts (HFSFs) were purchased from the Cell Resource Center, Institute of Basic Medical Sciences, CAMS/PUMC. The cells were cultivated in cell culture dishes of the 100 mm × 20 mm type. Dulbecco’s modified Eagle’s medium (DMEM, Corning Inc., Corning, NY, USA) was used in the dishes, along with 2 mM L-Glutamine Liquid (Beijing Solarbio, Science & Technology Co., Ltd., Beijing, China), 10% fetal bovine serum (FBS, Christchurch, New Zealand), and 1% penicillin–streptomycin solution (Gibco, Inc., Corning, NY, USA). Trypsin (Gibco, Inc., Corning, NY, USA) was used to digest and passage the cells after they had grown to confluence. The cells were kept in an incubator (Thermo Fischer Scientific, Waltham, MA, USA) at 37 °C with 5% CO_2_, and the culture medium was replaced every 48 h.

### 2.2. THz Sources and Radiation

**Figure 1:**
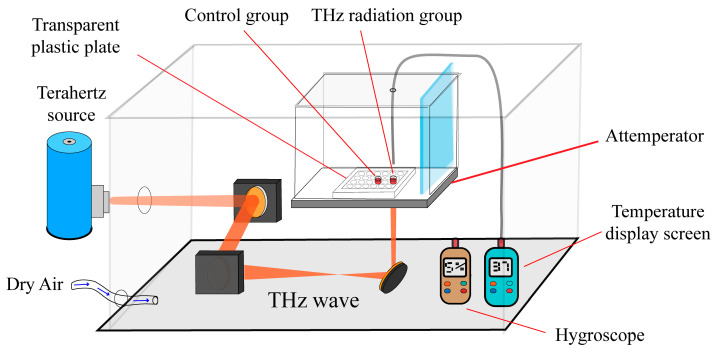
The schematic diagram of the terahertz radiation system. The terahertz waves emitted by the terahertz radiation source propagate through an optical system, forming a terahertz radiation spot that fully covers the HFSFs. The introduction of dry air mitigates terahertz wave attenuation induced by atmospheric moisture absorption. The metal radiation chamber is integrated with a heating system to provide an optimal growth environment for HFSFs. Blue arrows denote the flow direction of incoming dry air.

Terahertz quantum cascade lasers (THz-QCLs, Institute of Semiconductors, Chinese Academy of Sciences, Beijing, China) operated at frequencies of 3.10 THz (power: 0.26 mW) and 3.75 THz (power: 0.28 mW), respectively. The optical route system for transmitting terahertz waves is made up of a reflector and two off-axis parabolic mirrors (OPAs). The terahertz beam emitted by the radiation source is collected by the front off-axis parabolic mirror. A dual off-axis parabolic mirror optical system is adopted to collimate the beam and suppress divergence. The collimated parallel terahertz beam is focused by the rear off-axis parabolic mirror, yielding a tightly focused terahertz spot. By integrating the Golay detector (TYDEX, GC-1P) with a metal aperture, the position of the 1.6 cm diameter spot at the end of the terahertz beam can be determined. The terahertz beam was then directed by a reflector to propagate vertically upward, ultimately irradiating the HFSFs located at the bottom of the 24-well culture plate. The 24-well and 48-well culture plates (Corning Inc., Corning, NY, USA) used in this study were made of polystyrene. Previous studies have confirmed that polystyrene exhibits extremely low terahertz loss, with a refractive index ranging from 1.017 to 1.022 and a dielectric constant of 2.45 to 2.65 [35,36]. These excellent optical properties make it an ideal substrate for terahertz detection and related applications. HFSFs were exposed to terahertz radiation environments with average power densities of 0.13 mW/cm^2^ (corresponding to a frequency of 3.10 THz) and 0.14 mW/cm^2^ (corresponding to a frequency of 3.75 THz). The terahertz radiation chamber’s temperature was kept at 37 °C. The ambient humidity of the terahertz wave optical route system must be kept at 10% or lower to avoid significant attenuation of the terahertz waves during transmission. The schematic diagram of the entire terahertz radiation apparatus that we employed for this investigation is displayed in Figure 1.

### 2.3. Hypoxic Cell Culture System

**Figure 2:**
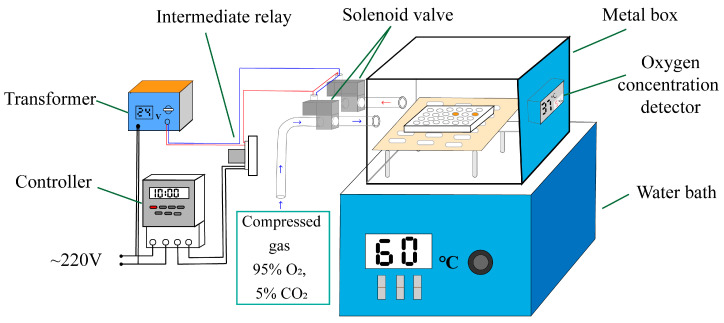
The schematic diagram of the hypoxic cell culture system. The gas control system regulates the output of the mixed gas (95% N_2_, 5% CO_2_) to reduce the O_2_ concentration inside the metal chamber while maintaining the internal pressure consistent with the external environment. The metal chamber, in conjunction with the water bath, provides a stable in vitro growth environment for HFSFs. Blue arrows indicate the inflow direction of the compressed gas mixture (95% N_2_ and 5% CO_2_), and red arrows indicate the outflow direction of the internal atmosphere.

After seeding the 24-well culture plates with a cell density of 2.0 × 10^4^ cells/well, the plates were incubated for 12 h at 37 °C, 21% O_2_, and 5% CO_2_. For a 12 h hypoxia induction experiment, the plates were then moved to a metal box chamber with 1.5–3% O_2_ at 37 °C. Within the metal box, the oxygen concentration detector (SWEVY, Guangzhou, China, SW-9820) was used to measure the temperature and O_2_ concentration. When the O_2_ content rises above 3%, a mixture of 95% N_2_ and 5% CO_2_ is contained in the metal box. Mostly, a controller (SHUGUANG, Shanghai, China, SG101D) works with two solenoid valves (Chint, Wenzhou, China, N2V025-08) and a small intermediate relay (Chint, Wenzhou, China, JZX-22F) to fill the gas mixture. During the gas-filling operation, the controller is in charge of controlling the simultaneous initiation of the two solenoid valves. A plastic tube connects one of the solenoid valves to a gas mixture pressure vessel, which fills it with a high-pressure mixture of 95% N_2_ and 5% CO_2_ to release surplus oxygen from the metal box. In order to balance the gas pressure inside the metal box with the outside pressure, the other solenoid valve’s job is to release the gas inside the box to the outside. To parameterize the controller, the time needed to raise the oxygen content in the metal box from 1.5% to 3% and to lower it from 3% to 1.5% during inflation is computed once the box is sealed. The water bath (Sunne, Shanghai, China, SN-HWS-1DJ) was used to hold the metal box, and its temperature was adjusted to attain 37 degrees Celsius in the center of the metal box. The 24-well culture plate was then set on a plastic tray, whose height matches the oxygen concentration detector’s sensor position. This arrangement is intended to give the cells the best possible growing circumstances. After 12 h of hypoxic incubation, HFSF cells were immediately subjected to 120 min irradiation at 3.1 THz and 3.75 THz, respectively. A schematic diagram of the hypoxic cell culture system used in this study is shown in Figure 2.

### 2.4. Cell Viability Assay

Three independently cultivated biological samples were employed for each radiation exposure condition, with three technical replicates measured per sample. The average values of experimental and control groups were calculated separately. Briefly, cells were treated as follows: HFSF cell viability was assessed strictly in compliance with the guidelines provided in the Cell Counting Kit-8 (CCK-8, Meilunbio, Dalian, China, MA0218). Trypsin digestion was started whenever the cell fusion in the cell culture dish reached 70% to 80%. Cell viability assays were performed on HFSF cells exposed to 3.75 THz, with irradiation durations of 60 min, 120 min, and 180 min, respectively. The cells were then injected into 48-well growth plates. After that, 400 μL of complete culture medium was added to each well to bring the cell density down to 8 × 10^4^ cells/well. A total of 200 μL of total culture solution containing 10% CCK-8 reagent per well was added to the original culture solution after the terahertz radiation was finished. Finally, HFSFs were incubated for two hours in a cell incubator. In order to reduce measurement errors caused by the instrument, each sample’s absorbance (OD450) was measured three times at 450 nm using an EnSpire multimode plate reader (Bio-Rad, Songjiang, Shanghai, China). The blank control group had no cells and only complete medium and CCK-8 solution. The percentage of absorbance at 450 nm that separated the radiation group from the control group was used to assess cell viability. By comparing the absorbance percentage at 450 nm between the irradiation and control groups, cell viability was evaluated.

### 2.5. RNA Extraction and qPCR

Three biological replicates were prepared for each treatment group, and three technical replicates were performed for qPCR quantification of each sample. Total RNA was isolated from HFSFs using the total RNA extraction reagent (TRIzol, ABclonal, Wuhan, Hubei, China, RK30129) in accordance with the manufacturer’s instructions. A NanoDrop One Microvolume UV-Vis Spectrophotometer (Thermo Fischer Scientific, Waltham, MA, USA) was then used to measure the amount of extracted RNA. The GoScript^TM^ Reverse Transcription System (Promega, Madison, WI, USA, A5000) was used to synthesize cDNA. The study’s target genes were COL1A1, MMP-2, TIMP-2, and TGF-β2, while the internal control was Glyceraldehyde-3-phosphate dehydrogenase (GAPDH). Table 1 lists the primer sequences utilized for the gene-specific primers, which were created and purchased from Takara Bio (Dalian, China). The GoTaq qPCR Master Mix (Thermo Fischer Scientific, Waltham, MA, USA, A6001) and LightCycler^®^96 (Roche Diagnostics Applied Science, Mannheim, Germany) were used to conduct the qPCR analysis. A 10 min initial denaturation at 95 °C is followed by 40 cycles of denaturation at 95 °C for 15 s and annealing/elongation at 60 °C for 60 s in the qPCR reaction. The comparative threshold cycling (2^−ΔΔCq^) approach was then used to investigate the relative expression levels of the genes under examination [37]. RNA extraction and qPCR analysis were carried out in HFSF cells following exposure to 3.1 THz and 3.75 THz irradiation for 30 min, 60 min, and 120 min, respectively. After being exposed to terahertz radiation for 120 min, HFSFs were cultured for another 12 h in a cell culture incubator. These cells were used to examine the terahertz radiation’s delayed effects.

### 2.6. Proteomics Analysis

For proteomic analysis, three pooled biological replicates were generated for both the radiation-treated group and the control group. Fifteen 24-well cell culture plates were used, with one treated sample and one control sample harvested from each plate, resulting in 15 individual samples per group. Within each group, every five samples were equally pooled to form one replicate, yielding three pooled replicates for protein extraction and sequencing. To further validate the hysteresis effect of terahertz radiation on cellular functions, this study subjected HFSFs to repeated 3.75 THz radiation. HFSFs were seeded at a density of 1.6 × 10^5^ cells/well in 24-well culture plates, with the experimental and control groups arranged in an alternating well pattern. For each experimental well containing adherent HFSFs, 3.75 THz terahertz radiation was administered for 120 min per day over a consecutive 5-day period. After the completion of the terahertz radiation research, HFSFs were digested using trypsin (Gibco, Waltham, MA, USA), and the precipitated cells were then collected. The collected cell pellets were washed three times with ice-cold phosphate-buffered saline (PBS) and subsequently flash-frozen and stored on dry ice until proteomic analysis. The proteome was analyzed by Scale Biomedicine Technology Co., Ltd. (Beijing, China). HFSFs exposed to terahertz radiation were lysed using a denaturing buffer. The Bradford protein quantitative kit (Beyotime, Songjiang, Shanghai, China) protocol was followed in order to quantify the total protein concentration. After trypsin-gold (Promega, Madison, WI, USA, V5280) was used to digest these protein samples, they were slowly put onto a C18 deionization column to desalt them. Every sample that eluted was collected and lyophilized. Using an Orbitrap Astral mass spectrometer and the Vanquish^TM^ Neo UHPLC system (Thermo Fisher Scientific, Waltham, MA, USA), proteomic analyses were carried out on each sample. Spectronaut 17 software (Biognosys AG, Schlieren, Switzerland) was used to process and analyze the raw data of liquid chromatography–tandem mass spectrometry (LC-MS/MS) detection using the default parameters. Download the Uniprot/NCBI database’s Homo sapiens protein sequence database (FASTA).

The functional profiles and molecular characteristics of proteins in HFSF cells were determined via bioinformatics analysis. Differentially expressed proteins (DEPs) were annotated and functionally enriched based on Gene Ontology (GO) and Kyoto Encyclopedia of Genes and Genomes (KEGG) databases, with the aid of the DAVID bioinformatics resource (https://davidbioinformatics.nih.gov/, accessed on 28 December 2025). Using the Search Tool for Interacting Genes (STRING) database (https://cn.string-db.org/, accessed on 28 December 2025), a protein–protein interaction (PPI) network was constructed based on 213 DEPs (fold change [FC] ≥ 1.2, FC ≤ 0.83, false discovery rate [FDR] < 0.05) derived from HFSFs. The minimum required interaction score was set at 0.900 (corresponding to the highest confidence level), and disconnected nodes were hidden from the network. The Cytoscape software (version 3.7.0) was employed to quantify the node parameters and visualize the constructed protein–protein interaction (PPI) network. The degree of each node was calculated to define the size of the corresponding protein node in the network.

### 2.7. Statistics and Reproducibility

GraphPad Prism software (version 9.5.0, GraphPad Inc., San Diego, CA, USA) was used to statistically analyze all of the data. The mean ± standard deviation (SD) of at least three independent, repeated experiments is how the study’s data are presented. The homogeneity of variance between the radiation group and control groups, as well as between groups with varying radiation durations, was evaluated using Levene’s test. If Levene’s test yields *p* ≥ 0.05, the data satisfy the assumption of homogeneity of variance, and the independent samples Student’s *t*-test is adopted for intergroup comparisons. In contrast, Welch’s *t*-test was used if *p* < 0.05, rejecting the assumption of homogeneity of variance. Student’s *t*-test and Welch’s *t*-test are employed to determine if significant differences exist between two independent groups. Statistical significance was determined using the following criteria: Differences with *p* ≥ 0.05 were considered non-significant and are not reported; and *p* < 0.05 was considered statistically significant. The statistical importance of the results is clearly indicated by the *p*-value, which has the following significance levels: * *p* < 0.05, ** *p* < 0.01, *** *p* < 0.001, and **** *p* < 0.0001.

## 3. Results

### 3.1. The Effect of Terahertz Radiation on the Viability of HFSFs

**Figure 3:**
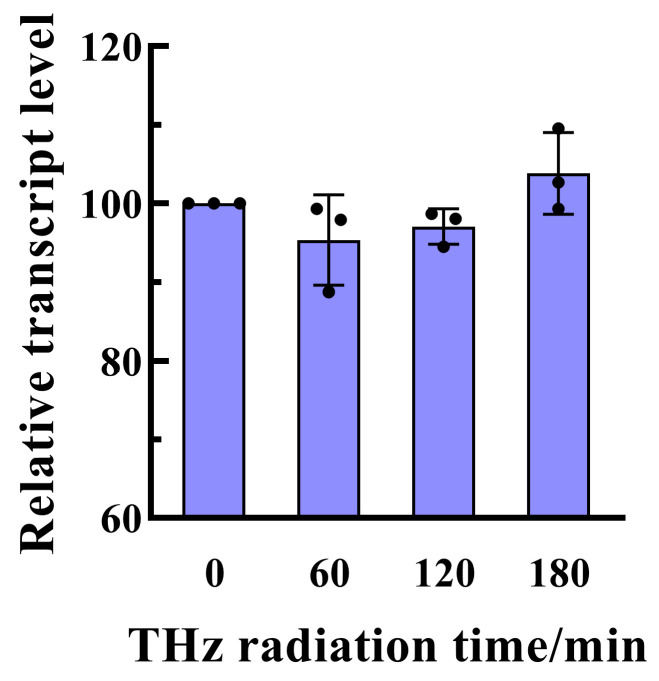
The effect of terahertz radiation on the viability of HFSFs. After irradiating HFSFs with 3.75 THz radiation for 60, 120, and 180 min, respectively, no statistically significant difference in cell viability was observed between the experimental and control groups. All experimental results are presented as the mean ± standard deviation (SD) (*n* = 3).

Variations in HFSF survival were investigated as the duration of 3.75 THz radiation increased. After being cultured for 12 h, HFSFs were subjected to terahertz radiation for 60, 120, and 180 min, respectively. Cell viability was then evaluated by CCK-8 testing. The viability of HFSFs was found to be unaffected significantly by 3.75 THz radiation (Figure 3).

### 3.2. Effects of 3.10 THz and 3.75 THz Radiation on COL1A1 Gene Expression in HFSFs

Scleral fibroblasts represent the core cellular component responsible for maintaining the structural and functional integrity of the sclera. Through the modulation of collagen metabolism, including synthesis and degradation, as well as extracellular matrix remodeling, these cells sustain the normal microstructure and mechanical homeostasis of the sclera. To investigate the effects of key physical parameters of terahertz radiation (exposure duration and frequency) on HFSF function, this study selected COL1A1 as a core detection index. After HFSFs were cultured for 12 h, the cells were subjected to 3.10 THz or 3.75 THz waves for 30, 60, or 120 min. Meanwhile, HFSFs were exposed to terahertz radiation for 120 min, and alterations in COL1A1 gene expression were evaluated following a subsequent 12 h of culture. This is used to elucidate the long-term effects of terahertz radiation, which is crucial for assessing its biosafety.

**Figure 4:**
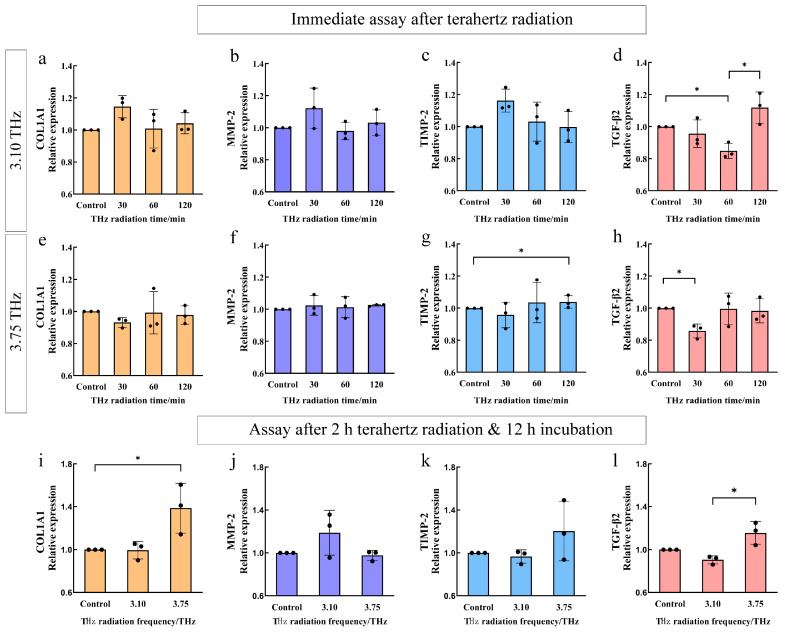
Effects of terahertz radiation on the expression of genes regulating collagen synthesis and degradation in HFSFs. Gene expression of COL1A1 (orange), MMP-2 (purple), TIMP-2 (blue) and TGF-β2 (red). Panels (**a**–**d**) present the gene expression results after 3.10 THz irradiation; panels (**e**–**h**) show the results after 3.75 THz irradiation; panels (**i**–**l**) illustrate the gene expression levels following 120 min of 3.10 THz and 3.75 THz irradiation, followed by a 12 h static incubation in the incubator. The mean ± standard deviation (SD) (*n* = 3) is used to represent all experimental results in this work, where * *p* < 0.05.

After 30 min of exposure to 3.10 THz radiation, COL1A1 gene expression rose (1.14 ± 0.04, vs. control), albeit this increase was not statistically significant. When compared to the control group, extended exposure times (60 and 120 min) produced insignificant effects (Figure 4a). After 12 h of exposure to terahertz radiation, there were no significant changes in COL1A1 gene expression in HFSFs (Figure 4h). Likewise, within 120 min of exposure to 3.75 THz radiation, no discernible alterations in expression were found (Figure 4e). Collectively, these findings demonstrate that exposure to 3.10 THz and 3.75 THz radiation exerts no significant impact on the expression of collagen metabolism genes in HFSFs within the 120 min observation window. However, HFSFs’ COL1A1 (1.39 ± 0.13, *p* < 0.05, vs. control) gene expression significantly increased after being exposed to 3.75 THz radiation (Figure 4h). This suggests that the effects of 3.75 THz radiation on HFSFs may involve a hysteresis effect.

### 3.3. Effects of 3.10 THz and 3.75 THz Radiation on MMP-2 and TIMP-2 Gene Expression in HFSFs

The TIMP-2 gene is the natural regulator of MMP-2, which can break down a range of collagens [38]. It has been shown that the renewal of the scleral outer matrix in mammalian models of myopia depends on the balance between MMP-2 and TIMP-2 [39]. As a result, distinct alterations in MMP-2 and TIMP-2 species gene expression were assessed in this investigation as indicators of collagen degradation. In comparison to controls, HFSFs exposed to 3.10 THz waves for 30 min showed increased expression of the MMP-2 (1.12 ± 0.07, vs. control) and TIMP-2 (1.16 ± 0.04, vs. control) genes; however, these increases were not statistically significant (Figure 4b and Figure 4c, respectively). In particular, it has been noted that low levels of TIMP-2 encourage MMP-2 activation [40]. The interaction between MMP-2 and TIMP-2 may be the cause of the observed phenomena. However, after 30 min of continuous terahertz radiation, this slight upregulation of MMP-2 and TIMP-2 gene expression was no longer observed. After 12 h of exposure to terahertz radiation, HFSFs exposed to 3.10 THz radiation showed an increase in MMP-2 gene expression (1.19 ± 0.12, vs. control) (Figure 4j). Concurrently, no statistically significant alterations in TIMP-2 gene expression were observed (Figure 4k). However, neither gene expression showed statistical significance, which may be attributable to the limited number of experimental samples employed.

Exposure to 3.75 THz radiation for 120 min resulted in a statistically significant variation in TIMP-2 expression (1.04 ± 0.03, *p* < 0.05, vs. control) (Figure 4g); however, no consistent trend of upregulation or downregulation was evident. Concurrently, regarding other results during the 120 min exposure to 3.75 THz radiation, no significant changes in the expression of the MMP-2 and TIMP-2 genes were observed (Figure 4f and Figure 4g, respectively). These findings suggest that 120 min of exposure to 3.75 THz radiation exerted negligible effects on the expression of the MMP-2 and TIMP-2 genes in HFSFs. After 12 h of exposure to terahertz radiation, the TIMP-2 gene was upregulated in HFSFs (1.20 ± 0.16, vs. control) during exposure to 3.75 THz radiation (Figure 4k). There were no significant changes in MMP-2 gene expression (Figure 4j). However, neither gene expression showed statistical significance. Nevertheless, the findings suggest that delayed post-radiation assessment exhibits more pronounced regulatory effects on gene expression compared to immediate detection. This observation provides critical experimental evidence for optimizing key radiation parameters, specifically exposure duration and frequency, in subsequent studies.

### 3.4. Effects of 3.10 THz and 3.75 THz Terahertz Radiation on TGF-β2 Gene Expression in HFSFs

TGF-β is a key regulator of extracellular matrix renewal [41]. This study found distinct variations in the expression of the TGF-β2 gene, a hallmark of extra-scleral matrix remodeling. When HFSFs were subjected to 3.10 THz and 3.75 THz radiation at varying exposure durations, there was a downregulation of TGF-β2 (3.10 THz, 60 min: 0.85 ± 0.03, *p* < 0.05, vs. control; 3.75 THz, 30 min: 0.86 ± 0.02, *p* < 0.05, vs. control) gene expression (Figure 4d and Figure 4h, respectively). But when terahertz radiation exposure was prolonged to 120 min, 3.10 THz radiation caused the TGF-β2 (1.11 ± 0.06, *p <* 0.05, vs. 60 min radiation group) gene expression level in HFSFs to increase, while the TGF-β2 gene expression level in the 3.75 THz radiation-treated group returned to the normal control level. In HFSFs exposed to 3.10 THz radiation, the expression level of the TGF-β2 gene restored to normal control levels 12 h after the terahertz radiation exposure ended (Figure 4l). In contrast, there was a noticeable increase in the expression level of the TGF-β2 gene (1.16 ± 0.06, *p* < 0.05, vs. 3.10 THz 120 min radiation group) in HFSFs treated with 3.75 THz radiation. According to the experimental findings, HFSFs’ TGF-β2 gene is more sensitive to 3.10 THz radiation in response to terahertz radiation exposure, but 3.75 THz radiation causes a more noticeable hysteresis effect in controlling its gene expression.

### 3.5. Effects of 3.10 and 3.75 THz Radiation on Hypoxia-Induced Collagen Synthesis and Degradation in HFSFs

**Figure 5:**
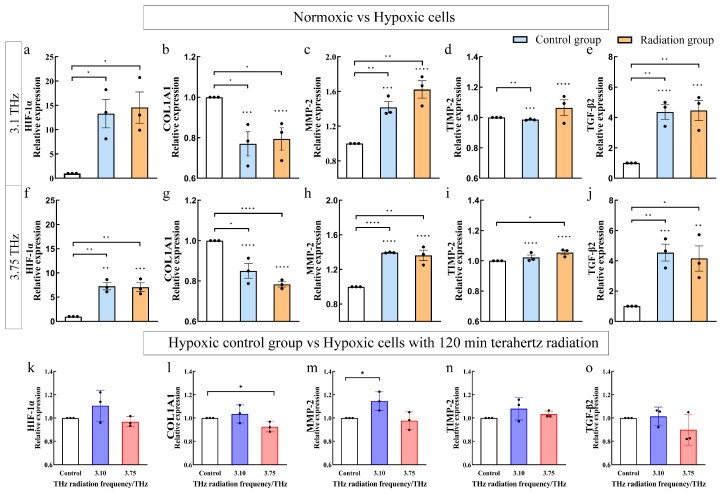
Effects of terahertz radiation on the expression of genes related to collagen synthesis and degradation in hypoxic HFSFs. (**a**–**e**) Comparison of gene expression between normoxic HFSF cells and hypoxic HFSF cells (hypoxic control group, hypoxic + terahertz irradiation group) upon 3.1 THz irradiation within a 120 min exposure period; (**f**–**j**) comparison of gene expression between normoxic HFSF cells and hypoxic HFSF cells (hypoxic control group, hypoxic + terahertz irradiation group) upon 3.75 THz irradiation within a 120 min exposure period; (**k**–**o**) comparison of gene expression between hypoxic HFSF cells treated with 3.1 THz or 3.75 THz irradiation for 120 min and their corresponding hypoxic control groups. From left to right, the panels display the expression levels of HIF-1α, COL1A1, MMP-2, TIMP-2, and TGF-β2, respectively. In this work, all experimental results are presented as mean ± standard deviation (SD) (*n* = 3), where * *p* < 0.05, ** *p* < 0.01, *** *p* < 0.001, and **** *p* < 0.0001.

Scleral hypoxia has recently been recognized as a major factor in the development of myopia. The main transcription factors that cause cells to adapt to hypoxia are called hypoxia-inducible factors (HIFs). In order to determine the hypoxic state of HFSFs, the study assessed the differential expression of the HIF-1α gene in these cells. After 12 h of hypoxia induction (1.5–3% O_2_), HFSFs were subjected to 3.10 and 3.75 THz waves for 120 min in a radiation device at 37 °C. According to the results of the experiment, HIF-1α (12.13 ± 4.70, vs. normal cultured cells) and MMP-2 (1.38 ± 0.08, vs. normal cultured cells) gene expression was upregulated after 12 h of hypoxia (Figure 5k and Figure 5m, respectively), while COL1A1 (0.80 ± 0.08, vs. normal cultured cells) gene expression was downregulated (Figure 5l). The results indicate that the myopia model cells built in this work are effective, even though the experimental data did not reach statistical significance.

This study analyzed the effects of terahertz radiation on hypoxia modeling of HFSFs. The expression of collagen synthesis and degradation genes was detected in hypoxic HFSFs exposed to radiation at 3.10 THz and 3.75 THz (Figure 5). The findings of the research indicate that 3.75 THz radiation significantly downregulates COL1A1 (0.92 ± 0.02, *p* < 0.05, vs. control) gene expression in hypoxia-induced HFSFs (Figure 5l). MMP-2 and TIMP-2 gene expression, however, did not alter significantly between the radiation group and the control group (Figure 5m and Figure 5n, respectively). This finding suggests that while 3.75 THz radiation may hinder collagen synthesis, it has almost no effect on collagen breakdown. In contrast, the expression of the MMP-2 (1.14 ± 0.05, *p <* 0.05, vs. control) gene was significantly increased in hypoxic HFSFs by 3.10 THz radiation, while the expression of the COL1A1 and TIMP-2 genes showed no significant effect. This finding suggests a potential relationship between 3.10 THz radiation and the acceleration of collagen degradation in HFSFs.

### 3.6. Alterations in Global Protein Expression of HFSFs Induced by 3.75 THz Repetitive Radiation

**Figure 6:**
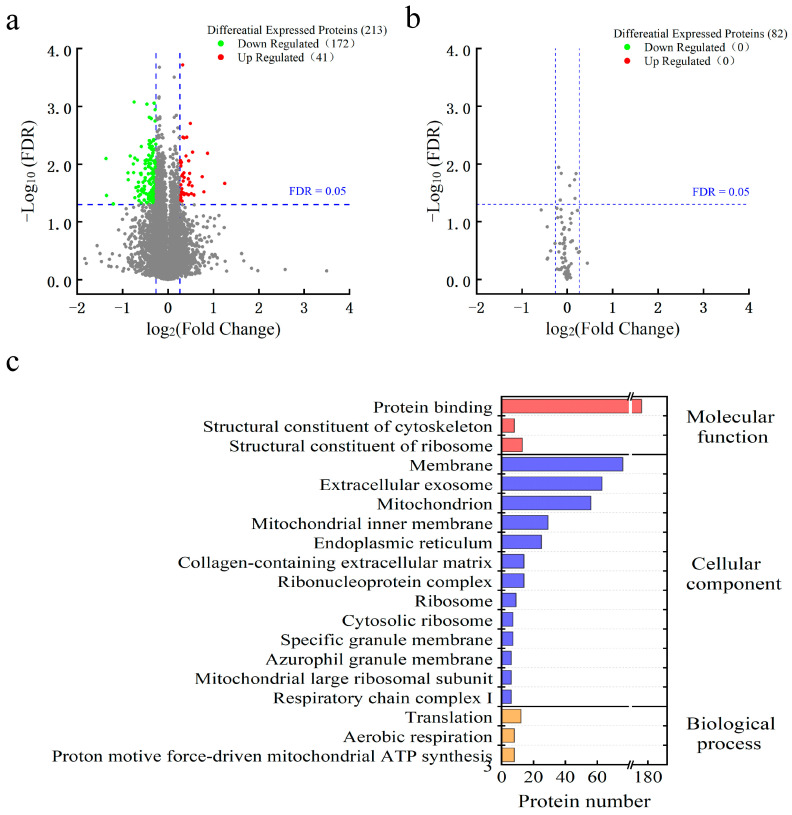
Effects of 3.75 THz repetitive radiation on the global proteomic expression profile of HFSFs. (**a**) Global protein expression in HFSF cells exposed to terahertz radiation is depicted by the volcano plot. Vertical dashed lines mark fold change (FC) thresholds of 0.83 and 1.2, and the horizontal dashed line indicates the false discovery rate (FDR) cutoff of 0.05. Red dots represent upregulated proteins, while green dots denote downregulated proteins. (**b**) The expression of heat shock proteins (HSPs) in HFSF cells exposed to terahertz radiation is displayed in the volcano plot. The GeneCards database provided 82 heat shock protein data points (correlation score > 18) that were compared to the proteins identified in this study; (**c**) DEPs were subjected to GO analysis, which yielded a total of 3 MF terms (red), 3 BP terms (orange), and 13 CC terms (blue).

This study presents a preliminary exploration of the molecular effects of terahertz radiation on scleral fibroblasts. Exposure to 3.1 THz and 3.75 THz radiation within 120 min did not induce significant changes in the transcript levels of COL1A1, MMP-2, and TIMP-2 in normoxic HFSF cells. A trend toward elevated MMP-2 expression was observed at 12 h post 3.1 THz irradiation, although this change did not reach statistical significance. Under hypoxic culture conditions, this identical 120 min irradiation regimen markedly upregulated MMP-2. These results suggest that 3.1 THz irradiation may raise the risk of excessive collagen degradation in HFSF cells. A total of 12 h after 3.75 THz irradiation, alterations in COL1A1 and TGF-β2 levels were more pronounced than those observed immediately post-irradiation. Molecular-level perturbations induced by terahertz radiation require time to propagate through complex protein interaction networks before stabilizing into a distinct biological phenotype. To comprehensively assess the global proteomic response of HFSFs to this delayed effect, we selected an excitation frequency of 3.75 THz and extended the post-irradiation incubation period to 22 h. Recognizing that prolonged incubation might allow cellular homeostatic mechanisms to mask radiation-induced alterations, we implemented a fractionated irradiation protocol. The number of exposures was limited to five cycles, determined by cell confluence in 24-well plates; exceeding this limit would require subculturing, which could introduce confounding procedural variables. Accordingly, proteomic analysis was performed immediately after the fifth irradiation cycle. The objective of the present study was to elucidate the mechanism underlying the effects of 3.75 THz repetitive radiation on HFSFs. Deps were screened based on the analysis of cellular proteomics datasets, followed by the performance of systematic functional annotation and enrichment analysis using bioinformatics approaches. Proteomics technology identified a total of 8151 proteins (Table S1). Using a stringent screening criterion set that included an FC ≥ 1.2, FC ≤ 0.83, and FDR ≤ 0.05 for DEPs, a total of 213 DEPs were identified. Of these DEPs, 41 were upregulated, accounting for 19.25% of the total, whereas 172 were downregulated, accounting for 80.75% (Figure 6a). These results indicate that 3.75 THz repetitive radiation induces significant alterations in the global proteomic expression profile of HFSFs. Furthermore, this study investigated the effects of 3.75 THz repeated radiation on the expression characteristics of heat shock proteins in HFSFs. This study retrieved 10,142 heat shock-related proteins from the GeneCards database (Version 5.26, https://www.genecards.org/, accessed on 8 January 2026), from which 122 proteins with a relevance score of no less than 18 were further screened. These candidate proteins were subsequently compared against the full-proteome dataset identified via proteomics techniques in the present study, leading to the final identification of 82 heat shock-related DEPs (Figure 6b) (Table S2). Analysis results indicate that among the 82 heat shock-related proteins identified through screening, only DNAJC6 exhibited an FC of 1.36, while the fold changes for all other proteins remained below 1.2. These findings suggest that exposure to 3.75 THz repeated radiation induces negligible heat stress effects in HFSFs.

To annotate the functional characteristics of differentially expressed proteins, Gene Ontology (GO) enrichment analysis was conducted, encompassing biological process (BP), molecular function (MF), and cellular component (CC) categories. GO analysis indicates that these differentially expressed proteins are primarily associated with ribosomal and mitochondrial functions in HFSFs (Figure 6c) (Table S3). Ribosome function-related terms primarily covered two CC (ribonucleoprotein complex and cytosolic ribosome), one BP (protein translation), and one MF (structural constituent of ribosome). Mitochondrial function-related entries primarily covered three cellular components (mitochondrial inner membrane, mitochondrial large ribosomal subunit, respiratory chain complex I) and two biological processes (aerobic respiration and proton motive force-driven mitochondrial ATP synthesis). Furthermore, five keratin members (KRT2, FC = 1.22; KRT9, FC = 0.80; KRT14, FC = 1.33; KRT16, FC = 2.38; KRT6A, FC = 1.39) and three tubulin members (TUBA1C, FC = 0.82; TUBA4A, FC = 0.60; TUBB3, FC = 0.75) were found to be enriched in the molecular function category structural constituent of cytoskeleton. Taken together, our results demonstrate that terahertz radiation potentially regulates the core biological functions associated with ribosomes, mitochondria, and cytoskeletons in HFSFs.

**Figure 7:**
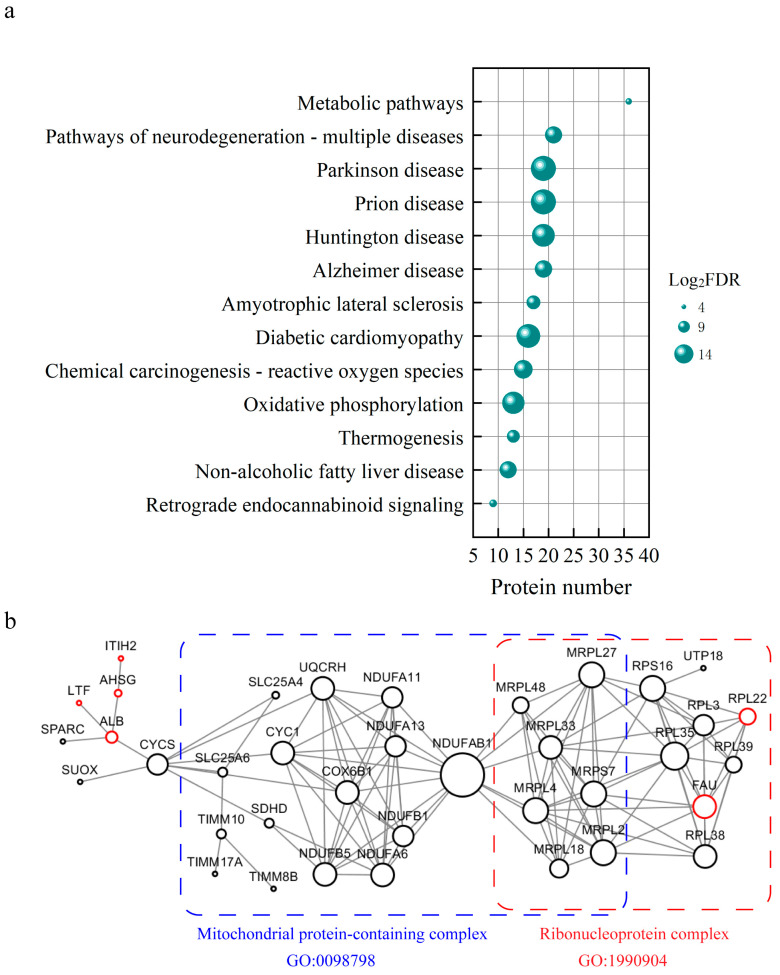
KEGG and PPI analyses of DEPs in HFSFs under 3.75 THz repetitive radiation. (**a**) KEGG pathway enrichment analysis of differentially expressed proteins. Signaling pathways with FDR < 0.05 were selected. (**b**) PPI network analysis results of differentially expressed proteins. Minimum required interaction score ≥ 0.9. Node size was proportional to the degree value. Red nodes represent upregulated proteins, and black nodes represent downregulated proteins. Blue boxes indicate differentially expressed proteins associated with the mitochondrial protein-containing complex, whereas red boxes indicate those associated with the ribonucleoprotein complex.

To gain deeper insights into how differentially expressed proteins modulate the signaling pathways in HFSFs, we conducted KEGG enrichment analysis (Figure 7a) (Table S4). The results of the KEGG enrichment analysis indicate that exposure to 3.75 THz repetitive radiation activates multiple signaling pathways associated with mitochondrial function, such as oxidative phosphorylation, pathways of neurodegeneration—multiple diseases, and chemical carcinogenesis—reactive oxygen species. These signaling pathways simultaneously encompass multiple mitochondrial respiratory chain complex proteins, including NADH-ubiquinone oxidoreductase or complex I (NDUFA6, NDUFAB1, NDUFB1, NDUFB5, NDUFA11, and NDUFA13), succinate dehydrogenase or complex II (SDHD), ubiquinone-cytochrome c oxidoreductase or complex III (UQCRH), and cytochrome c oxidase or complex IV (CYCS, COX6B1, CYC1).

Concurrently, KEGG enrichment analysis revealed that exposure to 3.75 THz repetitive radiation activates signaling pathways associated with ribosomal function. Further analysis of the PPI network demonstrated that the DEPs identified in this study could be categorized into two functional modules, namely mitochondrial ribosomal proteins and cytosolic ribosomal proteins (Figure 7b). Mitochondrial ribosomal proteins identified herein primarily include MRPL2, MRPL4, MRPL18, MRPL27, MRPL33, MRPL48 and MRPS7. The ribosome-related DEPs primarily included RPL3, RPL22, RPL38, RPL39 and RPS16. Among these proteins, only RPL22 exhibited an upregulated expression pattern, with a fold change (FC) of 1.40. The expression of these mitochondrial respiratory chain complexes and mitochondrial ribosomal proteins was downregulated following exposure to 3.75 THz repeated radiation.

### 3.7. Effects of 3.75 THz Repetitive Radiation on Collagen Metabolism in HFSFs

**Figure 8:**
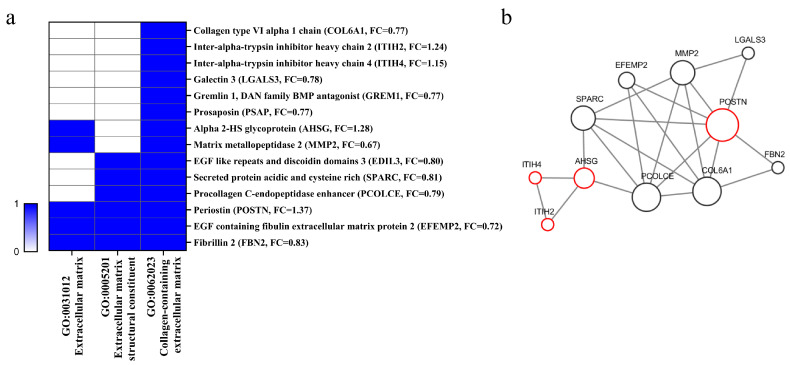
Effect of 3.75 THz repetitive radiation on the expression of proteins related to collagen synthesis and degradation in HFSFs. (**a**) Heatmap of proteins related to the extracellular matrix signaling pathway in scleral fibroblasts based on GO analysis. The vertical axis represents protein names and their fold changes, while the horizontal axis denotes extracellular matrix-related terms identified by GO functional annotation clustering. (**b**) Protein–protein interaction (PPI) network analysis of proteins enriched in the extracellular matrix signaling pathway of HFSFs. Minimum required interaction score ≥ 0.4. Node size was proportional to the degree value. Red nodes represent upregulated proteins, and black nodes represent downregulated proteins.

HFSFs represent the predominant cell population resident in the scleral tissue, which directly mediates the remodeling of the scleral extracellular matrix by precisely orchestrating the dynamic balance between collagen synthesis and degradation. In the results of GO enrichment analysis, 14 proteins associated with the collagen-containing extracellular matrix category under the CC ontology were significantly enriched. To further analyze the effects of 3.75 THz repetitive radiation on collagen metabolism in HFSFs, we employed the “Functional Annotation Clustering” module within GO analysis to perform cluster analysis on DEPs. The 213 DEPs were grouped into nine clusters. Among these, Cluster 5 exhibited enrichment in terms associated with the extracellular matrix, with a cluster enrichment score of 2.18. Of the significantly enriched ontology terms, two belonged to the CC category (i.e., collagen-containing extracellular matrix and extracellular matrix), whereas one was assigned to the MF category, namely extracellular matrix structural constituent. Figure 8a depicts the set of DEPs that were enriched in these three terms, as well as the magnitude of their fold changes. Analysis of the protein–protein interaction (PPI) network results identified a total of 11 interacting proteins, among which four were upregulated and seven were downregulated (Figure 8b). Notably, five proteins, including POSTN, PCOLCE, COL6A1, SPARC, and MMP2, exhibited high interaction degrees within the network, functioning as core hub nodes mediating interprotein crosstalk.

### 3.8. 3.75 THz Repetitive Radiation Inhibits the Activation of the TGF-β Signaling Pathway

**Figure 9:**
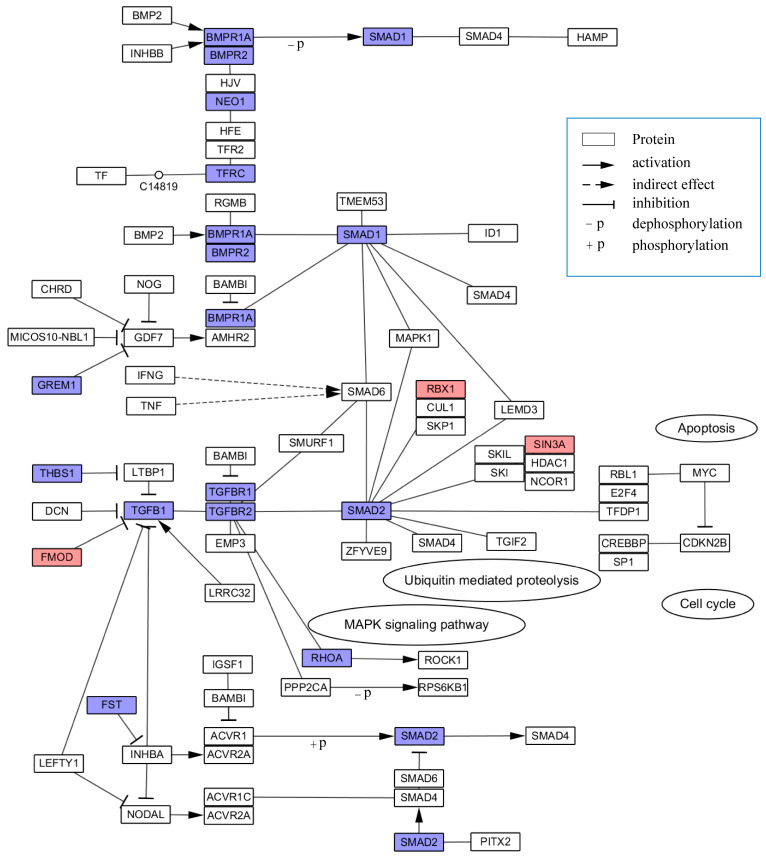
Visualization analysis of TGF-β signaling pathway-related proteins in HFSFs. Red: significantly upregulated (*p* < 0.05); blue: significantly downregulated (*p* < 0.05).

To further dissect the regulatory effects of repetitive 3.75 THz irradiation on the TGF-β signaling pathway, we performed enrichment visualization analysis of DEPs enriched within this cascade (Figure 9). KEGG enrichment analysis of the whole proteome dataset obtained upon repetitive 3.75 THz irradiation was performed via the DAVID database. Bioinformatic results revealed significant enrichment of the TGF-β signaling pathway (FDR = 0.03), in which 59 differentially expressed proteins were annotated to this pathway. Next, we downloaded the canonical TGF-β pathway (hsa04350) blueprint (85 constituent proteins) from KEGG and visualized the network with Cytoscape software. Of all proteins quantified in the present proteomic profiling, 21 were successfully mapped to the canonical TGF-β signaling pathway, among which three were upregulated and 18 were downregulated. Notably, core regulatory proteins of the TGF-β signaling cascade, including TGFB1 (FC = 0.82, *p* = 0.02), TGFBR1 (FC = 0.90, *p* = 0.04), TGFBR2 (FC = 0.93, *p* = 0.02), and SMAD2 (FC = 0.88, *p* = 0.02), were downregulated. Collectively, these results indicate that repetitive 3.75 THz irradiation may inhibit the activation of the TGF-β signaling pathway.

## 4. Discussion

Many biomolecules, including proteins and DNA, have vibrational and rotational energy levels that fall within the terahertz wave range [42,43]. Terahertz radiation at specific frequencies may cause structural and activity changes in biomolecules, according to a variety of theoretical studies and laboratory findings [44,45]. For example, Wu et al. demonstrated that 44.0 THz radiation triggers vibrational resonance in purine moieties connected via weak hydrogen bonds, rupturing inter-base-pair hydrogen bonds and markedly accelerating double-stranded DNA unwinding [46]. The same group later experimentally confirmed that 35.2 THz irradiation efficiently facilitates duplex unwinding during DNA origami assembly [47]. Cheon et al. reported that 1.7 THz exposure induces global DNA demethylation in hematological tumor cells, underscoring the therapeutic potential of terahertz radiation at this frequency for cancer treatment [48]. In studies focusing on structural alterations of proteins caused by terahertz radiation, 3.67 THz irradiation of bovine serum albumin (BSA) induced measurable intensity shifts in characteristic spectral signatures in both UV absorption and circular dichroism (CD) spectra. Yamazaki et al. reported that terahertz radiation at 0.46 THz exerts modulatory effects on the conformation of actin protein [49]. Long-term exposure to 3.1 THz terahertz radiation significantly accelerates the aggregation of Aβ42 monomers and inhibits the fibrillization stage of Aβ42 oligomers [50]. Homenko et al. demonstrated that 100 GHz radiation triggers conformational changes in alkaline phosphatase (ALP) and markedly reduces enzymatic activity via non-thermal mechanisms [51]. Collectively, these findings reveal that terahertz radiation can effectively modulate the structural conformation of biomacromolecules, and such structural alterations may further trigger morphological or functional changes at the cellular level. The majority of recent experimental investigations on terahertz radiation parameters have not found any appreciable rises in temperature or modifications in the expression of heat shock proteins [52,53,54]. These alterations are not the same as those seen with traditional heating. Terahertz radiation can affect the activity and function of cells and organisms through non-thermal mechanisms, according to the body of evidence. Global gene expression analysis of biological cells and tissues utilizing microarray and RNA sequencing techniques has become a potent research tool with the introduction of genomics technology, offering vital support for elucidating the mechanisms behind the impacts of terahertz radiation. A systematic evaluation and meta-analysis of the non-thermal effects of terahertz radiation was carried out by Dione et al. [55]. In more than a dozen published studies, the response of gene expression to terahertz radiation was analyzed. The findings show a significant degree of heterogeneity, according to the results, which could be related to variations in the irradiation circumstances and cell types that were chosen. Meanwhile, a number of genes exhibited either overexpression or underexpression across all experiments involving terahertz radiation. Initial studies have demonstrated that 0.10 THz can affect HFSF activity and COL1A1 gene expression while simultaneously blocking TGF-β pathway activation. Recent research endeavors have indicated that fluctuations in pivotal radiation parameters, such as frequency, power, and exposure duration, have the potential to elicit alterations in the comprehensive genome-wide expression profile. To further investigate the potential medical applications of terahertz radiation, this study examined the effects of 3.10 THz and 3.75 THz radiation on the expression of collagen synthesis and degradation-related genes (COL1A1, MMP-2, TIMP-2, and TGF-β2) in scleral fibroblasts under varying irradiation durations. Furthermore, we compared the gene expression profiles immediately obtained post-irradiation with those from delayed assessments. This provides some empirical support for the selection of key physical parameters in research into the biological effects of terahertz radiation. Integration of proteomics and bioinformatics analyses enabled the identification and correlation of 14 key proteins involved in the remodeling of the scleral extracellular matrix. These findings provide significant molecular evidence for further investigation into the regulatory mechanisms by which terahertz radiation modulates collagen metabolism in the sclera.

To date, there are no reports on the effects of terahertz radiation on scleral fibroblasts. However, the rational selection of detection indicators is crucial for elucidating the biological effects of terahertz radiation on these cells and for screening key radiation parameters, such as frequency, power, and exposure time. The sclera was formerly thought to be a tissue type that was comparatively metabolically inactive in earlier research. According to recent studies, the sclera is a dynamic tissue that may continuously renew itself to control the refractive state of the eye [2,56]. Collagen makes up the majority of the scleral tissue in mammals, with type I collagen predominating [4,13,57]. It has been shown that type I collagen metabolism influences the mechanical characteristics of the sclera, which in turn influences scleral axial elongation and the development of myopia [58,59]. The main cellular component of the sclera, scleral fibroblasts, may identify alterations in the extracellular matrix around them. By continuously synthesizing and breaking down collagen, they contribute to the remodeling of the scleral extracellular matrix [60,61]. Consequently, this study selected COL1A1, a gene associated with collagen synthesis, and MMP-2 and TIMP-2, genes associated with collagen degradation, as biomarkers to investigate the effects of terahertz radiation on scleral fibroblasts at the molecular level. This study first examined the impact of various durations of terahertz radiation on the expression of three target genes. These preliminary results suggest that 120 min of terahertz radiation in the 3.10 THz and 3.75 THz frequency ranges may not directly alter the expression of these three genes. Based on our previous observation that significant gene expression differences persisted 16 h after irradiation [25], we speculate that structural and conformational changes in biomolecules may only become detectable following transmission through complex biomolecular networks and cascade amplification. In order to test this hypothesis, HFSFs were subjected to 120 min of terahertz radiation and then continued to be cultured (incubated) for a further 12 h after the irradiation in order to analyze gene expression. The results demonstrated that, while there were no significant differences in the expression of MMP-2 and TIMP-2, the COL1A1 gene exhibited significant upregulation. These findings provide preliminary support for our hypothesis that conformational changes in biomolecules induced by terahertz radiation may require a certain time delay and can be gradually accumulated and amplified through complex signal transduction networks, thereby eliciting responses at the level of cellular gene expression. This may provide a plausible explanation for why significant effects were observed only when detection was performed following a delay after radiation exposure. As this study is intended as a preliminary exploration, with the primary aim of confirming the feasibility of using terahertz radiation to modulate the function of scleral fibroblasts, the dose–response relationship between the radiation parameters and the expression of the three genes has not yet been systematically elucidated. Future research is urgently needed to clarify the following: first, whether extending the duration of radiation exposure (e.g., from 4 to 12 h) directly enhances the magnitude of the gene expression response; second, whether different durations of radiation exposure all induce significant changes within the 12 h recovery period following irradiation; and third, whether this radiation-induced regulatory effect is long lasting—that is, whether it persists at 24 h, 48 h, or later after irradiation. The clarification of these key spatiotemporal parameters is of great significance for the establishment of the optimal terahertz radiation therapy regimen, and it is also a key focus of our future research.

In the current study, radiation at frequencies of 3.10 THz and 3.75 THz was found to induce alterations in TGF-β2 gene expression. These alterations point to a possible intimate relationship between terahertz radiation and TGF-β signaling pathway activation. Kim et al. have found that fs-THz radiation can activate the TGF-β signaling system in the skin of mice. Consequently, this has the effect of impeding the healing process of wounds [16]. In the Shang et al. study, Caenorhabditis elegans was exposed to 0.263 THz radiation, which resulted in a decrease in the expression of numerous epidermal collagen genes regulated by the TGF-β signaling system [62]. Although the exact mechanism behind this occurrence is yet unknown, certain genes in the TGF-β signaling pathway showed enrichment in a different study conducted on mice with terahertz radiation-induced arthritis. In our previous investigation, proteomic analysis of HFSFs exposed to 0.10 THz radiation revealed reduced expression levels of several proteins linked to the TGF-β signaling pathway [22]. The pleiotropic regulatory protein TGF-β controls a wide range of biological activities, such as cell division, migration, proliferation, death, and extracellular matrix formation [63]. In the canonical TGF-β signaling pathway, activated TGFB1 binds to the cell surface receptor TGFBR2, which subsequently triggers the activation of TGFBR1. This process initiates the phosphorylation of downstream SMAD2/3 proteins, enabling phosphorylated SMAD2/3 to form a heteromeric complex with SMAD4. The assembled SMAD complex then translocates into the nucleus and, in cooperation with nuclear co-factors, modulates the transcription of TGF-β-responsive target genes. In the present study, the core proteins of the TGF-β signaling pathway, including TGFB1, TGFBR1, TGFBR2, and SMAD2, were significantly downregulated. The TGF-β signaling system may be highly sensitive to terahertz radiation, according to preliminary results. This needs more verification, though, in a variety of biological systems and under a wider range of terahertz radiation settings (such as radiation frequency, power density, and duration).

To further elucidate the underlying mechanism by which the hysteresis effect of terahertz radiation modulates the biological behaviors of HFSFs, we performed a proteomic analysis on HFSFs subjected to five rounds of 3.75 THz radiation exposure. Our experimental data revealed that 3.75 THz radiation had minimal impact on the cellular viability of HFSFs; notably, no substantial upregulation of heat shock proteins was observed, nor was the activation of critical apoptotic signaling cascades detected. These observations collectively support the conclusion that repetitive exposure to 3.75 THz radiation does not elicit prominent thermal effects in HFSFs. Systematic analysis of the entire set of proteins quantified by proteomic assays revealed 213 differentially expressed proteins, with 172 proteins exhibiting decreased expression and 41 proteins showing increased expression. Collectively, these data support the notion that 3.75 THz radiation regulates protein expression patterns in HFSFs through non-thermal effects. GO enrichment analysis results indicated that these DEPs are mainly localized to ribosomes and mitochondria in HFSFs. Meanwhile, PPI network analysis revealed that the core DEPs involved in the interaction network are primarily components of mitochondrial protein-containing complexes and ribonucleoprotein complexes. Ribosomes represent the primary sites for protein synthesis within cells [64]. In this study, the expression levels of multiple ribosomal subunit proteins were found to be significantly downregulated. On the basis of these findings, a hypothesis is proposed that the suppression of ribosomal subunit protein expression may be the key factor responsible for the substantial proportion of downregulated differentially expressed proteins—as high as 80%—observed in the terahertz radiation-treated group. KEGG pathway enrichment identified robust activation of the “Pathways of neurodegeneration—multiple diseases” signaling cascade. PPI analysis illustrated that the differential proteins mapped to this pathway predominantly modulate mitochondrial function, with abundant components belonging to mitochondrial respiratory complexes I to IV. Such mitochondrial complexes constitute the central functional machinery supporting oxidative phosphorylation (OXPHOS) [65]. During oxidative phosphorylation (OXPHOS), these respiratory complexes mediate electron transport and establish a proton electrochemical gradient to store energy. Complex V (ATP synthase) then harnesses this gradient to power ATP production [66]. There is a close regulatory relationship among complexes I–IV, and a functional abnormality in any of these key complexes can lead to mitochondrial dysfunction. Downregulation of these differentially expressed proteins (DEPs) implies that 3.75 THz irradiation suppresses OXPHOS activity in HFSFs, which further results in attenuated ATP synthesis, impaired respiratory chain function, and mitochondrial dysfunction. Impairment of OXPHOS complexes inherently triggers the generation of reactive oxygen species (ROS) [67], with complexes I and III being the primary sites of damage. Basal ROS levels exert physiological signaling roles and enable adaptive responses to mild cellular stress. In contrast, an overaccumulation of ROS exceeding the buffering capacity of cellular antioxidant defenses disrupts mitochondrial ATP biosynthesis, provoking energy metabolic dysfunction and promoting neurodegenerative pathogenesis. This chronic pathological process unfolds across years, causing progressive structural and functional deterioration of mitochondria and the entire cell. Consistent with the above evidence, our KEGG enrichment results revealed significant activation of signaling pathways associated with classic neurodegenerative disorders, including Alzheimer’s, Huntington’s, and Parkinson’s disease, further supporting this regulatory mechanism. The mitochondrial cascade hypothesis of Alzheimer’s disease proposes that accumulated oxidative damage reduces ATP content and elevates oxidative stress, ultimately eliciting Aβ toxicity [68]. This event further forms a vicious feedback loop that drives the development of neurodegenerative lesions. Furthermore, although the exact etiology of Parkinson’s disease (PD) has not been fully elucidated, mitochondrial dysfunction, especially impaired Complex I activity, is widely recognized as a key pathogenic contributor. However, current findings on terahertz radiation and Alzheimer’s disease show conflicting results. For instance, Zhang et al. further validated that a three-month intervention with 0.14 THz radiation rescued cognitive impairment and alleviated AD-associated neuropathological alterations in Alzheimer’s disease mice [33]. Wu et al. reported that 14-day 33 THz irradiation effectively ameliorated cognitive deficits and restored neural homeostasis in dementia mouse models [34]. In addition, Shang et al. found that 0.1 THz irradiation markedly improved locomotor activity and associative learning ability in tau-transgenic nematodes. Mechanistically, THz treatment reduced tau protein aggregation and upregulated the expression of neuroprotective SKN-1 and DAF-16. However, contradictory findings regarding the biological effects of THz irradiation have also been reported [69]. For example, Wang et al. demonstrated that 3.1 THz radiation significantly promotes the nucleation and aggregation of Aβ42 monomers while inhibiting the fibrillization of Aβ42 oligomers. Consistently, their further study revealed that 3.1 THz exposure markedly increases intracellular ROS levels in Aβ-expressing CL4176 Caenorhabditis elegans, further verifying that 3.1 THz radiation exacerbates Aβ-mediated neurotoxicity. Nevertheless, current studies on the association between terahertz radiation and AD lack in-depth exploration of the intrinsic mechanisms by which THz irradiation induces mitochondrial dysfunction. Based on quantitative proteomic profiling, the present study provides novel insights into the regulatory mechanism underlying THz radiation-modulated mitochondrial function. However, further verification is still required to determine whether this change persists in nerve cells. A growing body of studies focusing on the biological effects of terahertz radiation has corroborated a close association between this type of radiation and mitochondria- and ribosome-associated proteins [24,70]. The regulatory role of these mitochondria- and ribosome-associated proteins in modulating the physiological functions of scleral fibroblasts remains unclear. Collectively, the experimental findings of this study provide preliminary insights into delineating the molecular mechanisms underlying the regulatory effects of terahertz radiation on mitochondrial and ribosomal functions.

GO functional enrichment analysis revealed that a total of 14 differentially expressed proteins were enriched in terms associated with the collagen-containing extracellular matrix. Thinning of the scleral tissue and diminished biomechanical qualities are the main features seen when myopia progresses [71]. Changes in the ratio of collagen synthesis to breakdown are linked to significant scleral thinning. This mainly entails decreased glycosaminoglycan content, elevated MMP-2 activity, and downregulated COL1A1 expression [72]. MMP-2 has been identified as a pivotal regulator of collagen degradation. The present study found that repeated exposure to 3.75 THz radiation significantly downregulated MMP-2 protein expression levels, suggesting that terahertz radiation at this frequency band may inhibit collagen degradation. COL6A1 has been detected in scleral tissues derived from human donors [73,74]. In a tree shrew myopia model, Gao et al. [75] demonstrated a significant reduction in COL6A1 mRNA levels in the sclera after a 4-day recovery period. However, the specific functional role of COL6A1 protein in scleral extracellular matrix remodeling remains to be fully elucidated. In a multi-omics integrative study, Hui et al. [76] identified PCOLCE as a key regulator of myopia and pathological myopia, closely associated with collagen stability and the biomechanical properties of the sclera. The present study has demonstrated that 3.75 THz repetitive radiation induces downregulation of MMP-2, COL6A1, and PCOLCE protein expression. To date, no studies have reported on the biological function of the AHSG protein within scleral tissue or its potential mechanisms. However, in proteomic analyses of aqueous humor samples from patients with high myopia, Du et al. [77] observed a significant upregulation of AHSG expression; notably, the expression levels of this protein were positively correlated with the subjects’ axial length. Zhao et al. [78] demonstrated in a murine form-deprivation myopia (FDM) model that scleral periostin (POSTN) overexpression drives fibroblast-to-myofibroblast transdifferentiation (FMT), which in turn contributes to the onset and progression of myopia. The present study demonstrates that repetitive radiation at 3.75 THz induces the upregulation of AHSG and POSTN protein expression in scleral fibroblasts. The present results serve as direct evidence for the impact of terahertz radiation on scleral collagen remodeling; however, its holistic regulatory effects on the process of scleral extracellular matrix remodeling have not been clearly defined. Accordingly, further research is warranted to perform an in-depth analysis of collagen metabolic processes, scleral thickness dynamics, and biomechanical characteristics using a more extensive panel of myopia animal models.

This study contains a number of limitations. The findings of this study at the gene and protein levels merely provide molecular evidence. Further validation at the scleral tissue level is required to confirm whether the upregulation of COL1A1 genuinely promotes collagen synthesis and whether the downregulation of MMP-2 effectively reduces collagen degradation. In order to better establish the dose–response relationship between terahertz radiation and alterations in the scleral extracellular matrix, it is necessary to increase the number of radiation cycles. The cumulative effects of prolonged irradiation require further evaluation in future studies. Additionally, the physical mechanisms linking terahertz radiation to alterations in intracellular proteins remain largely uncharacterized. In view of the present paucity of high-frequency terahertz radiation sources, the present study was conducted in such a manner as to restrict exploratory observations to the 3.75 THz frequency. As relevant technologies advance, future multi-frequency studies are expected to further deepen our understanding of the frequency specificity of this biological effect.

Beyond the above observations, this study mainly characterizes the regulatory effects of terahertz irradiation on cellular collagen expression, which corroborates the capacity of THz radiation to modulate biological macromolecules. Prior investigations have demonstrated that the non-thermal effects of THz waves can remodel the spatial conformation of biomacromolecules. Specifically, THz exposure regulates gene expression by altering DNA breathing dynamics, DNA methylation and double-strand unwinding, and also modulates the aggregation behavior of serum albumin, actin and amyloid proteins. The present study’s GO enrichment analysis revealed that numerous differentially expressed proteins are significantly enriched in functional categories including protein binding, RNA binding, and GTP binding, as well as mRNA metabolism and magnesium ion-associated biological processes. Collectively, these proteomic results provide robust molecular evidence to support the universal regulatory activity of THz radiation on biomacromolecules, further enriching the fundamental theoretical basis for understanding the biological responses triggered by terahertz irradiation.

## 5. Conclusions

This study investigated the effects of irradiation at 3.10 THz and 3.75 THz on the expression of genes and proteins associated with collagen metabolism in HFSFs. Neither of the two frequencies induced significant changes in the expression of collagen metabolism-related genes within a 120 min terahertz irradiation period. Furthermore, the effects of terahertz radiation persist for at least 12 h, with the expression levels of collagen-related genes being significantly higher at this time point compared to immediately post-irradiation. To further investigate this delayed effect, scleral fibroblasts were subjected to repeated terahertz radiation at 3.75 THz for five consecutive days, with a daily exposure duration of 120 min. Proteomic results indicate that repetitive 3.75 THz radiation induces the downregulation of various extracellular matrix and TGF-β signaling pathway proteins, potentially sharing an intrinsic correlation with the suppression of mitochondrial and ribosomal protein expression. This molecular-level evidence provides a crucial basis for elucidating the regulatory mechanisms of terahertz radiation on collagen metabolism functions in scleral fibroblasts.

## Figures and Tables

**Table 1:** qPCR primer sequences.

Gene	Forward Primer (5′–3′)	Reverse Primer (5′–3′)
GAPDH	GCACCGTCAAGGCTGAGAAC	TGGTGAAGACGCCAGTGGA
COL1A1	CCCGGGTTTCAGAGACAACTTC	TCCACATGCTTTATTCCAGCAATC
MMP2	CTCATCGCAGATGCCTGGAA	TTCAGGTAATAGGCACCCTTGAAGA
TGFB2	TTACACTGTCCCTGCTGCACTT	GGTATATGTGGAGGTGCCATCAA
TIMP2	GGAGCACTGTGTTTATGCTGGAA	GAGACATGCGCAGTCTGCTTG
HIF-1α	CCTAACGTGTTATCTGTCGCTTTGA	TCTCTGAGCATTCTGCAAAGCTA

## Data Availability

The original contributions presented in this study are included in the article/Supplementary Materials. Further inquiries can be directed to the corresponding author.

## Supplementary Materials

The following supporting information can be downloaded at: https://www.mdpi.com/article/10.3390/cells15141285/s1; Table S1: 3.75 THz repeated radiation proteomics data; Table S2: Differential heat shock proteins; Table S3: GO analysis results; Table S4: KEGG analysis results.

## Abbreviations

The following abbreviations are used in this manuscript:

HFSFs	Human fetal scleral fibroblasts
DEPs	Differentially expressed proteins
ECM	Extracellular matrix
HSPs	Heat shock protein
TGF-β	Transforming growth factor-β
COL1A1	Type I collagen
MMP-2	Matrix metalloproteinase 2
TIMP-2	Tissue inhibitor of matrix metalloproteinase-2
